# Supplementary material for: Extracellular vesicles from stem cells rescue cellular phenotypes and behavioral deficits in SHANK3-associated ASD neuronal and mouse models
Source: Cell Death Dis. 2026 Feb 22;17(1):244. doi: 10.1038/s41419-026-08474-x (PMC12966433; doi:10.1038/s41419-026-08474-x)
Supplement: Supplementary file 1 — Supplementary Information [file 41419_2026_8474_MOESM1_ESM.docx]

**SUPPLEMENTARY MATERIALS LEGENDS**

**Supplementary Table 1-** EV Protein Markers according to *MISEV*(47) guidelines for all four types of EVs isolated.

**Supplementary Table 2-**  The list of proteins identified for all four types of EVs isolated.

**Supplementary Figure 1-** (a) Immunocytochemistry images of the individual channels of PKH-67 labelled EVs (green), MAP2 (red), and DAPI (blue) showing the uptake of EVs by both neuronal (MAP2+) and non-neuronal cells. Scale bar, 20 µM. (b) The full blot for the western hybridisation performed for CD63 for Control neuron EVs, SHANK3 neuron EVs, and iPSC EVs. (c) Ponceau S staining for the total protein from the three EVs is also presented.

**Supplementary Figure 2-** (a) Immunocytochemistry images of the individual channels of CTIP2 (red), MAP2 (orange), and DAPI (blue) for (i) untreated control neurons, (ii) control neurons treated with EVs derived from SHANK3 mutant neurons, and (iii) control neurons treated with synthetic liposomes. The EVs were added at two time points (see methods) (The merged images are shown in Fig 2b); Scale bar, 20 µM. (b) The quantification of the CTIP2*+* neurons among MAP2*+* neurons in (i) untreated control neurons, and (ii) control neurons treated with EVs derived from SHANK3 neurons. *Data presented as mean±SD*

**Supplementary Figure 3-** (a) Immunocytochemistry images of CTIP2 (red), MAP2 (orange), and DAPI (blue) for untreated SHANK3 mutant neurons and SHANK3 mutant neurons treated with EVs derived from control cortical neurons. The EVs were added at two time points (see methods) (The merged images are represented in Fig 3a); Scale bar, 20 µM. (b) Immunocytochemistry images of the individual channels of CTIP2 (red), MAP2 (orange), and DAPI (blue) for untreated SHANK3 mutant neuron*s* and SHANK3 mutant neurons treated with MSC-derived EVs. The EVs were added three times during the differentiation (see methods) (The merged images are shown in Fig 4c); Scale bar, 20 µM.

**Supplementary Figure 4 -** (a) Immunocytochemistry images of the individual channels of CTIP2 (red), MAP2 (orange), and DAPI (blue) for untreated SHANK3 mutant neurons and SHANK3 mutant neurons treated with iPSC-derived EVs. The EVs were added three times during the differentiation (see methods) (The merged images are represented in Fig 5c); Scale bar, 20 µM. (b) The quantification of CTIP2*+* neurons among MAP2*+* neurons in SHANK3 mutant neurons, SHANK3 mutant neurons treated with EVs derived from control cortical neurons, SHANK3 mutant neurons treated with MSC-derived EVs, and SHANK3 mutant neurons treated with iPSC-derived EVs. *Data presented as mean±SD*

**Supplementary Figure 5-** (a) Zeta potential quantification by Zetasizer^TM^ using dynamic light scattering of MSC and iPSC*-*derived EVs (b) NTA (Nanosight NS300) video frames for EVs obtained from MSC and iPSCs. (c) Neurite analysis of control neurons, control neurons treated with SHANK3 neuron-EVs, SHANK3 neurons, SHANK3 neurons treated with control neuron-EVs, SHANK3 neurons treated with MSC-EVs, SHANK3 neurons treated with iPSC-EVs

**Supplementary Figure 6 -** (a) *SynGO* cellular and biological component pathways for EVs derived from control neurons showing enrichment for synaptic proteins. (b) Similarly, for EVs derived from SHANK3 mutant neurons show higher enrichment for synaptic proteins. (c) Similar plots for MSC-EVs show enrichment for synaptic protein, but less than neuron-derived EVs. (d) The iPSC-EVs SynGO plots do not show high enrichment for synaptic proteins exclusively.

**Supplementary Figure 7 -** (a) A volcano plot displaying the differentially expressed proteins identified between EVs derived from SHANK3 mutant neurons and MSC-derived EVs. (b) The biological process analysis of the differentially expressed proteins between EVs derived from SHANK3 mutant neurons and MSC-derived EVs. (c) A volcano plot displays the differentially expressed proteins identified between EVs derived from SHANK3 mutant neurons and iPSC-derived EVs. (d) The biological process analysis of the differentially expressed proteins between EVs derived from SHANK3 mutant neurons and iPSC-derived EVs.

**Supplementary Video 1**- A representative ESP test video of the WT ICR mouse. The mouse was presented to neutral and stressed mice (stimuli) for 5 minutes after a 15-minute habituation period (for details, see methods). The video has been sped up 5X times.

**Supplementary Video 2**- A representative ESP test video of the *Shank3* KO ICR mouse. Other details same as in Supplementary Video 1.

**Supplementary Video 3**- A representative video for the characterisation and viewing of EVs using NTA (Nanosight NS300).

**Supplementary Video 4**- A video showing intranasal administration of iPSC EVs to *Shank3* ICR mice. The intranasal treatment was started at P9-P10 days-old mice pups and was continued till P60, every other day. The ESP behavioral test was done after P60 (also see methods).

**Supplementary Video 5**- A representative ESP test video of the *Shank3* KO ICR mouse after intranasal iPSC EV treatment. Other details are the same as in Supplementary Videos 1 and 2.
