# Supplementary material for: Extracellular vesicles from stem cells rescue cellular phenotypes and behavioral deficits in SHANK3-associated ASD neuronal and mouse models
Source: Cell Death Dis. 2026 Feb 22;17(1):244. doi: 10.1038/s41419-026-08474-x (PMC12966433; doi:10.1038/s41419-026-08474-x)
Supplement: Supplementary file 2 — Supplementary Table 1 [file 41419_2026_8474_MOESM2_ESM.docx]

| **EV origin** | **Protein markers** |
| --- | --- |
| Control neuron EVs | ANXA2, ACTG1, LGALS3BP, COL*, TUBB*, GAPDH, FN1 |
| SHANK3 neuron EVs | B2M , CD81, PDCD6IP, LGALS3BP, ACTB, COL*, TUBB*, TGFB1,FN1 |
| MSC EVs | B2M, CD63, CD81, CHMP4A, NT5E, PDCD6IP,COL*, TUBB*,FN1 |
| iPSC EVs | ANXA1, DSG1, DSC1, GAPDH, FGF2 |
