# Supplementary material for: Extracellular vesicles from stem cells rescue cellular phenotypes and behavioral deficits in SHANK3-associated ASD neuronal and mouse models
Source: Cell Death Dis. 2026 Feb 22;17(1):244. doi: 10.1038/s41419-026-08474-x (PMC12966433; doi:10.1038/s41419-026-08474-x)

Supplementary Fig 1

(a)

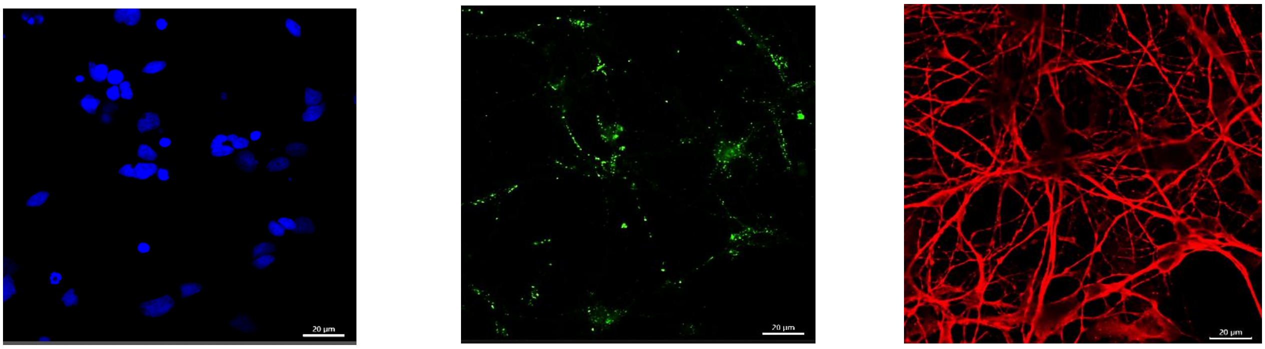

(b)

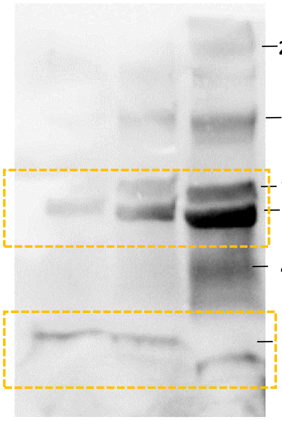

Full blot

Exposure time 4 seconds

(c)

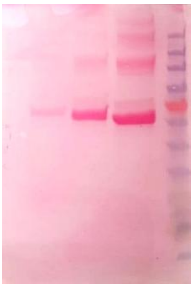

Ponceau S

Tris-glycine 4-20% gradient gel; PVDF membrane; BLUeye pre-stained marker(94964 Sigma-Aldrich)

Supplementary Figure 2

(a)

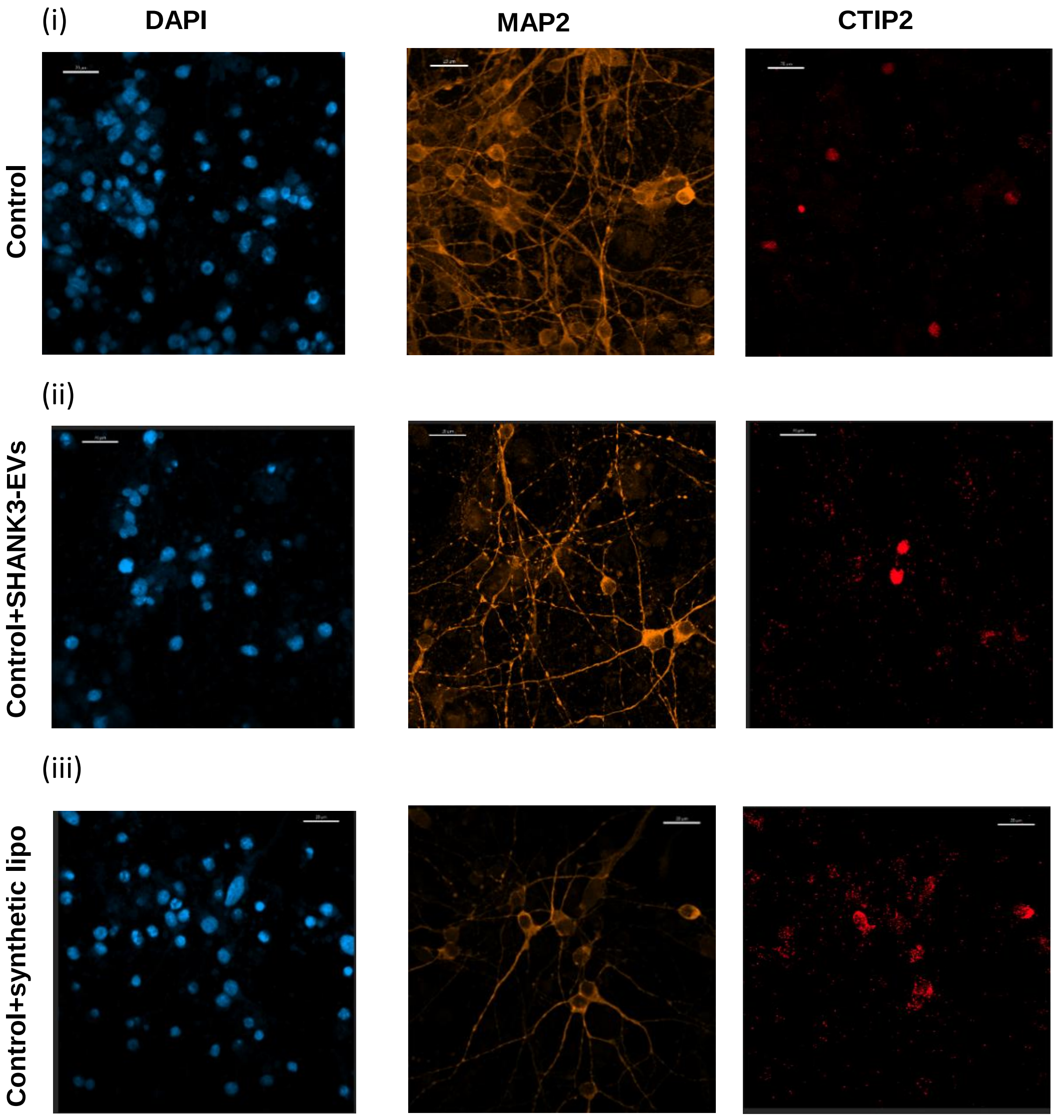

(b)

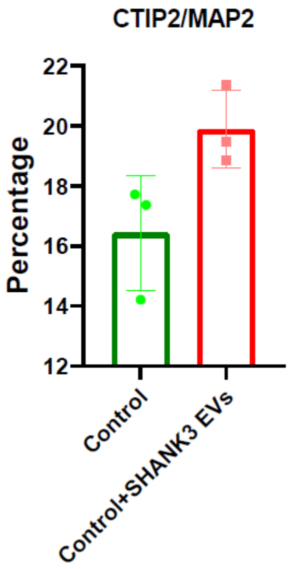

Supplementary Figure 3

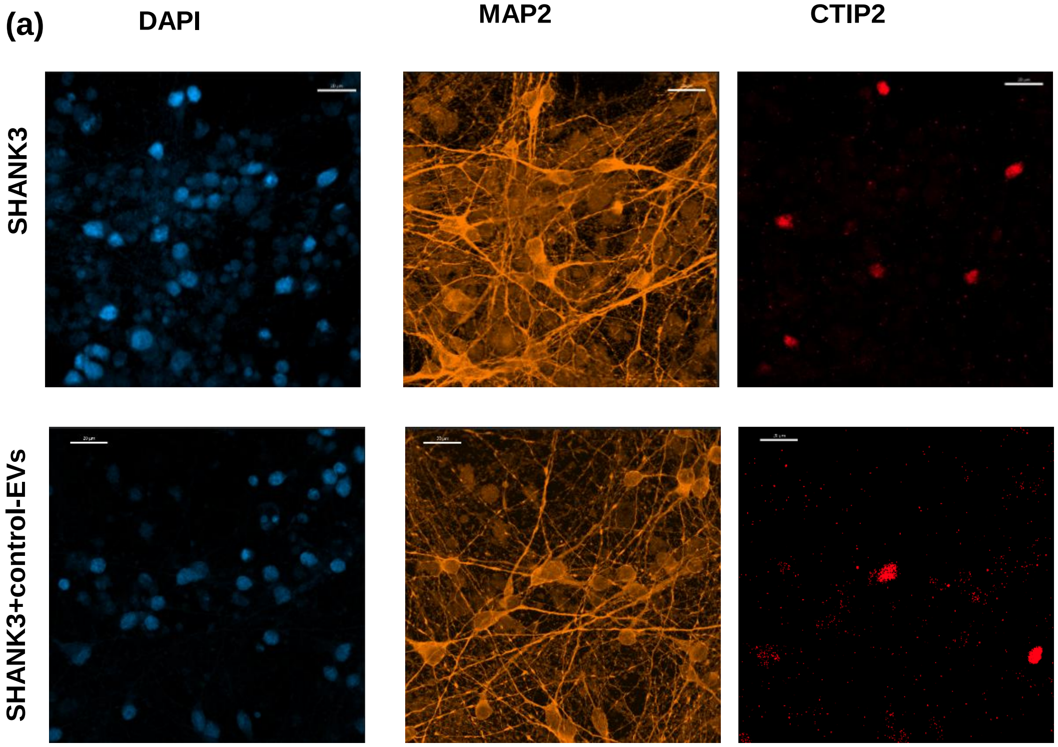

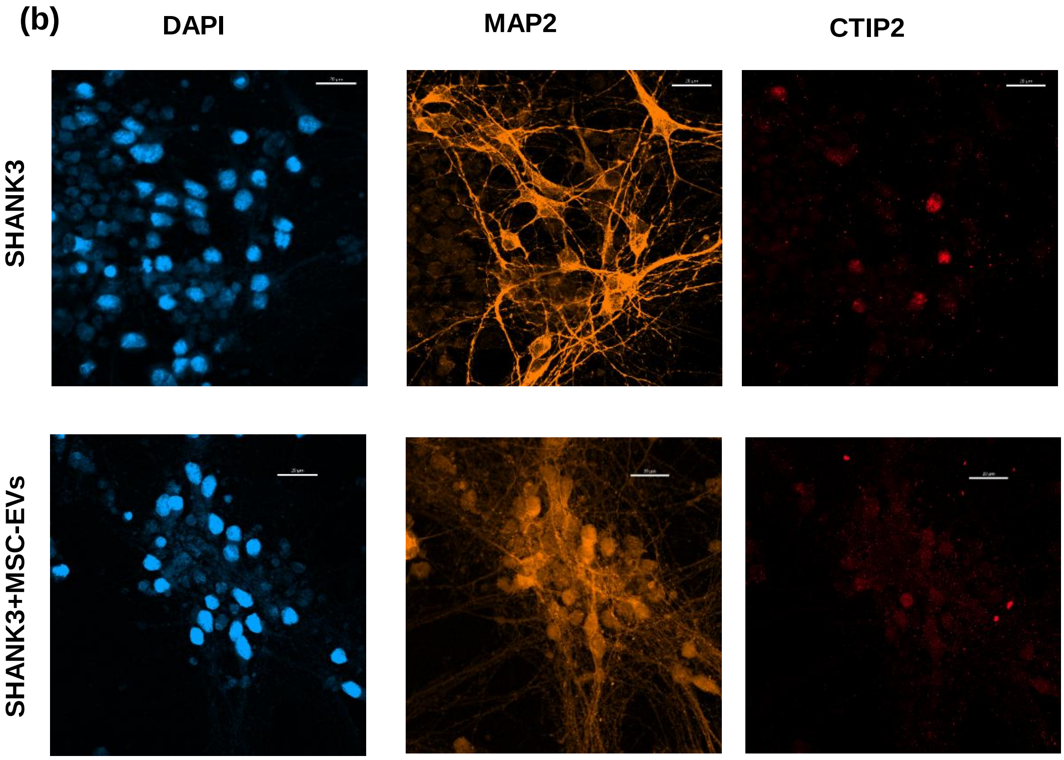

Supplementary Figure 4

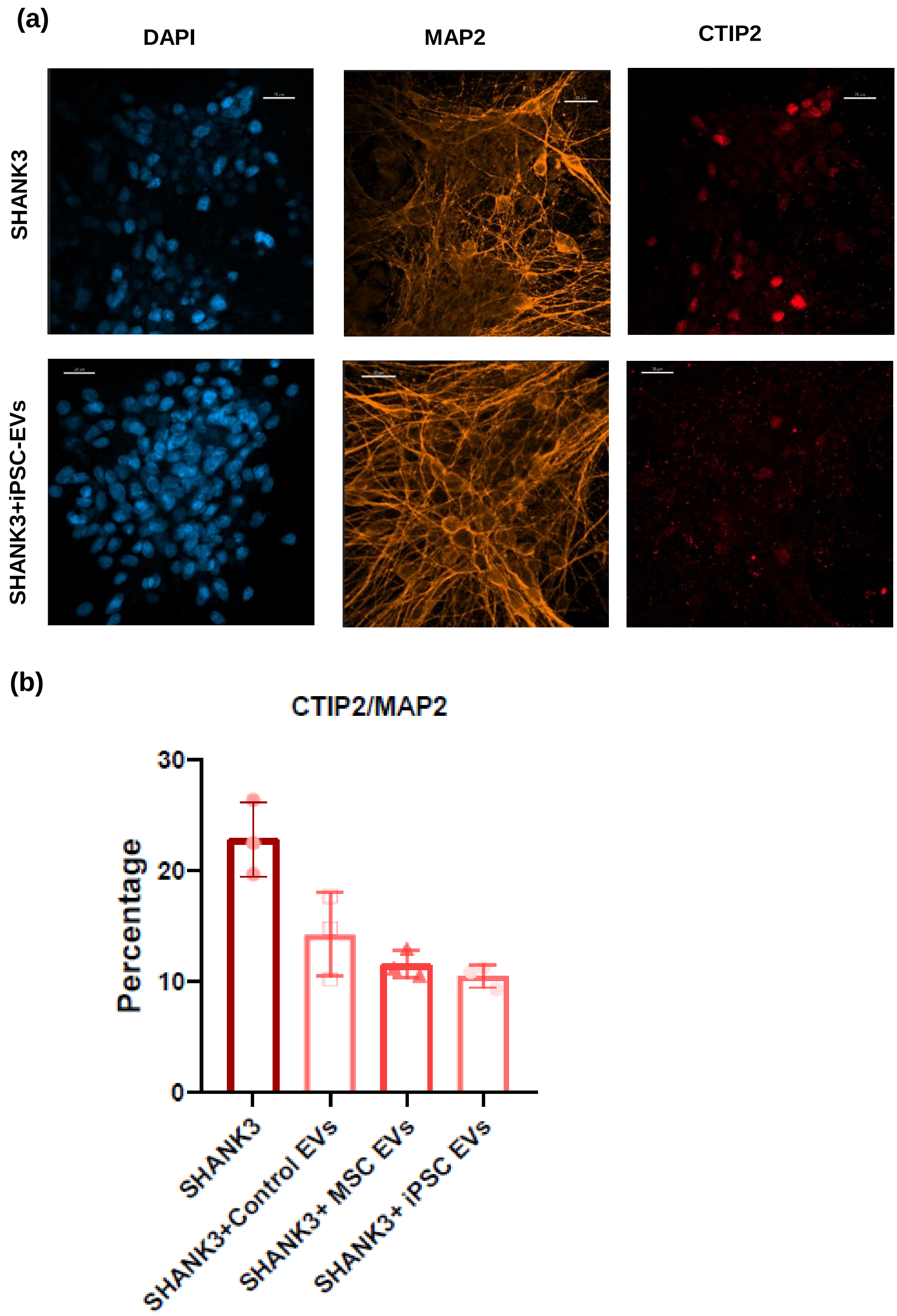

Supplementary Figure 5

(a)

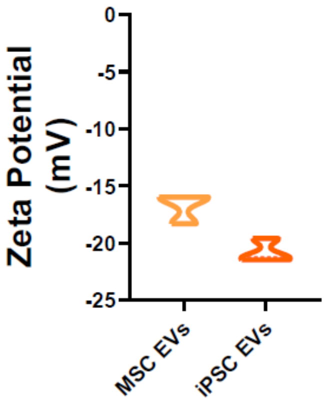

(b)

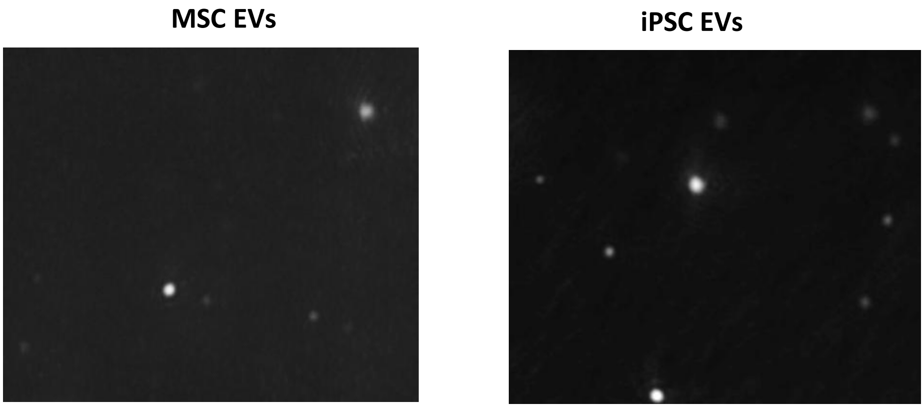

NTA video frame

(c)

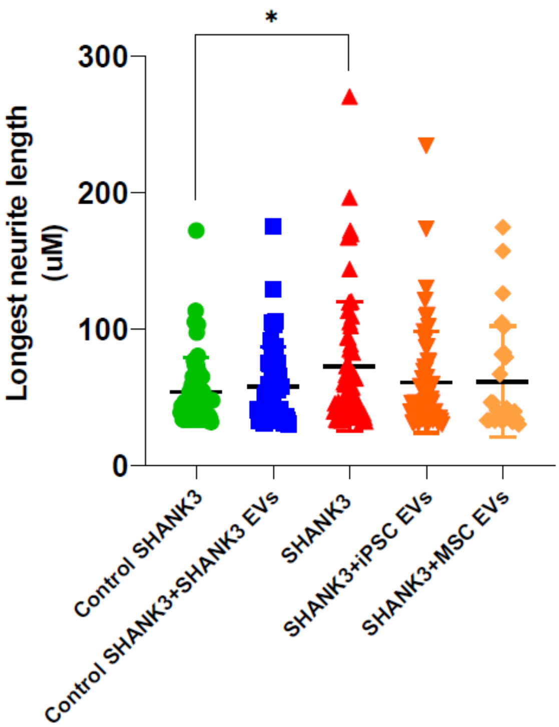

Supplementary Figure 6

(a) Control neuron EVs

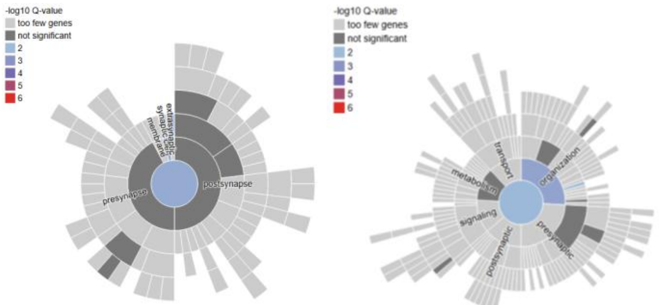

Cellular Component

Biological pathway

(b) SHANK3 neuron EVs

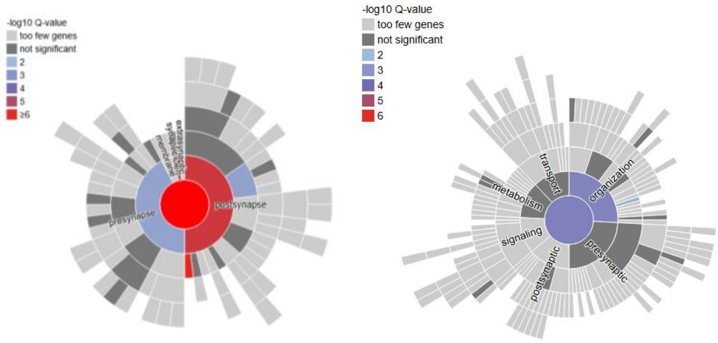

Cellular Component

Biological pathway

(c) MSC EVs

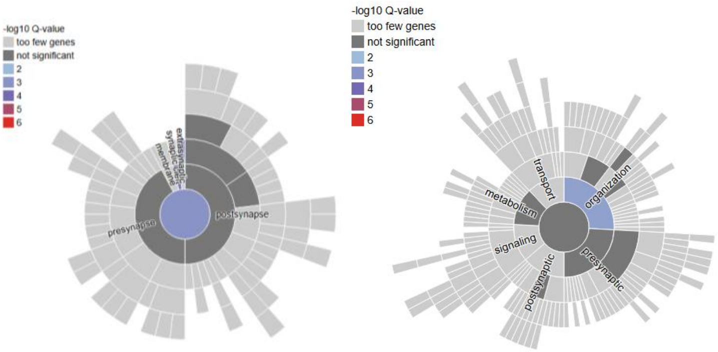

Cellular Component

Biological pathway

(d) iPSC EVs

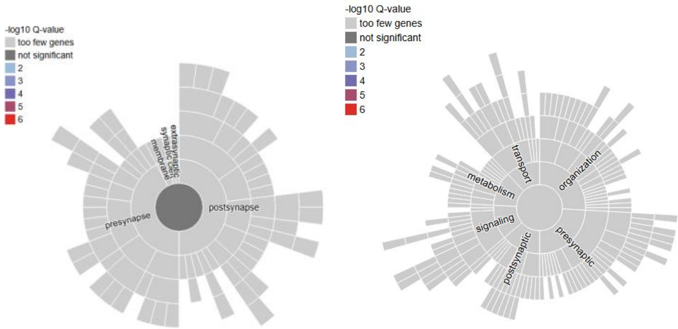

Cellular Component

Biological pathway

Supplementary Figure 7

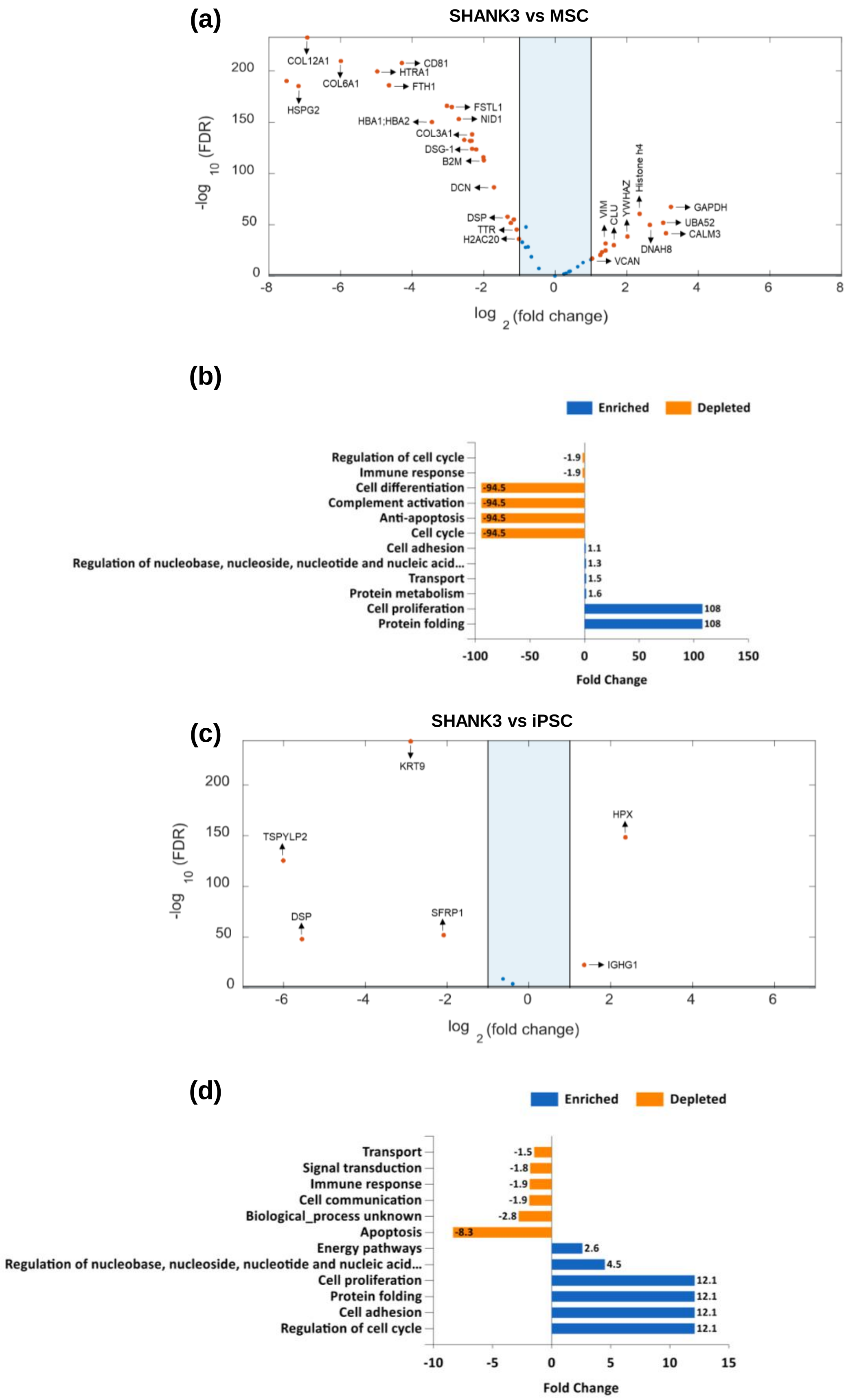

Supplement: Supplementary file 4 — Supplementary Figures 1–7 [file 41419_2026_8474_MOESM4_ESM.pdf]
